# A Cycle of Altered Proteasome and Reactive Oxygen Species Production in Renal Proximal Tubular Cells

**DOI:** 10.17140/tfmoj-4-128

**Published:** 2019-05-15

**Authors:** Nirmala Parajuli

**Affiliations:** Department of Pharmacology and Toxicology, University of Arkansas for Medical Sciences, Little Rock, AR 72202, USA

**Keywords:** Ubiquitin-proteasome system (UPS), Reactive oxygen species (ROS), Renal proximal tubular cells

## Abstract

**Aims:**

An intricate relationship exists between the mitochondrial function and proteasome activity. Our recent report showed in a rat model of renal transplantation that mitochondrial dysfunction precedes compromised proteasome function and this results in a vicious cycle of mitochondrial injury and proteasome dysfunction. In this study, we studied whether reactive oxygen species (ROS) has a role in proteasome alteration in renal cells and *vice versa.*

**Methods:**

We used the genomic and pharmacologic approach on rat normal kidney proximal tubular (NRK) cell lines. First, we knocked down β5 or Rpt6 subunit of the proteasome using small interfering RNA (siRNA) in NRK cells. We also treated NRK cells with Bortezomib, a proteasome inhibitor, and peroxynitrite (a potent ROS).

**Results:**

Studies with RNA interference showed increased mitochondrial ROS following knockdown of β5 or Rpt6 subunit in NRK cells. Similarly, pharmacological inhibition of the proteasome in NRK cells using Bortezomib also showed an increase of mitochondrial ROS in a dose-dependent manner. Next, exposing NRK cells to different concentrations of peroxynitrite provided evidence that the higher levels of peroxynitrite exposure decreased the key subunits (β5 and α3) of the proteasome in NRK cells.

**Conclusion:**

Our results suggest that proteasome inhibition/downregulation increases ROS, which then impairs proteasome subunits in renal proximal tubular cells.

## INTRODUCTION

The proteasome is the main machinery of the ubiquitin-proteasome system (UPS), which is essential for maintaining protein quality in all eukaryotic cells.^1–3^. The proteasome is composed of a cylindrical 20S proteasome and one or two 19S regulatory particle (s), both of which participate in selectively degrading ubiquitin-tagged proteins. The 20S proteasome has 3 to 7 protease active sites (β-catalytic subunits) that hydrolyze peptide bonds in chymotrypsin (β5)-, trypsin (β2)-, or caspase (βl)-like fashion.^2^ A functional proteasome plays a crucial role in degrading modified, misfolded, or damaged proteins to maintain intracellular protein homeostasis in kidneys. Therefore, any alteration to its components has the potential to disrupt protein homeostasis and could lead to pathological consequences.^4–6^

Excessive ROS generation is implicated in the pathogenesis of ischemia-reperfusion-induced renal damage.^7^ Studies suggest that ROS play a complex role in modulating proteasome activity. However, the role of the proteasome pathway during renal ischemia-reperfusion needs to be fully elucidated.^8^ Interestingly, *in vitro* data show that ROS exposure to mammalian cells can inhibit the proteasome function and can alter its composition.^9,10^ We recently demonstrated that mitochondrial dysfunction precedes compromised proteasome function in a rat model of renal cold storage plus transplantation, and reported the existence of a functional interdependent relationship between the proteasome activity and mitochondrial function in rat kidneys/renal cells.^11^ The main goal of this study was to examine a relationship between ROS and proteasome alteration in renal proximal tubular cells. Using normal rat kidney proximal tubular cell line (NRK), here, we demonstrate that proteasome inhibition increases mitochondrial ROS and exogenous ROS treatment declines proteasome subunit level.

## METHODS

### Cell Culture

Normal rat kidney proximal tubular cell line cultures (NRK-52E; ATCC No. CRL-1571) a.k.a. NRK cells were used in this study. The cells were maintained in growth medium (DMEM plus 5% fetal calf serum and 1% penicillin/streptomycin) and 5% CO_2_ incubator at 37 °C as described by the American Type Culture Collection (ATCC).

### Cell Treatment

NRK cells were seeded a day before small interfering RNA (siR-NA) transfection or Bortezomib or peroxynitrite treatment

a) siRNA transfection: NRK cells were transiently transfected with sigenomeβ5 (PSMB5) siRNA SMART pool or Rpt6 (PSMC5) siRNA SMART pool (100 nM) (Dharmacon, USA) using siRNA transfection reagent (Invitrogen, USA) in OP-TI-MEM (Invitrogen, USA) for 24 hours at 37 °C (as suggested by the manufacturer). A similar concentration of scrambled siRNA (Dharmacon, USA) was used as a control. The next day, cells were either harvested for protein extract or evaluated for ROS production (see MitoSOX™ Red fluorescence).
b) Bortezomib treatment: Bortezomib (BTZ) is a specific inhibitor of the β5 subunit of the proteasome.^12,13^ NRK cells were treated with BTZ (0, 10, 20, and 50 nM for 4 hr; Selleckchem, USA) in the normal growth medium. NRK cells treated with the same concentration of DMSO (no BTZ) were used as vehicle control. After 4 hrs, cells were evaluated for ROS production (see MitoSOX™ Red fluorescence).
c) Peroxynitrite treatment: Growth medium was removed, NRK cells were washed with PBS (pre-warmed at 37 °C), treated with peroxynitrite (30 or 300 μM; Calbiochem, USA) in warm PBS (37 °C) for 20 minutes. After 20 minutes, the PBS was removed, and normal growth medium added to the cells and cultured for 4 hr. NRK cells treated with the same volume of degraded peroxynitrite were used as vehicle control.

### Reactive Oxygen Species (MitoSOX™ Red Fluorescence) Measurement

MitoSOX™ Red reagent (Invitrogen Molecular Probes, USA) is a fluorogenic dye specifically targeted to mitochondria in live cells. Oxidation of MitoSOX™ Red reagent by superoxide produces a bright red fluorescence.NRK cells were preloaded with Mito-SOX™ Red reagent (5 μM, Molecular Probes, USA) for 10 min prior to Bortezomib treatment or siRNA transfection (against β5 or Rpt6 subunit). After 4 hrs of BTZ treatment or 24 hrs of siRNA transfection, growth medium from NRK cells was replaced with warm PBS. Red fluorescence was then visualized using a Nikon Eclipse E800 microscope with a rhodamine filter using a water immersion objective (60X). All images were captured with equal exposure times. Fluorescence intensity of the captured image was evaluated using Image J software. Corrected total cell fluorescence (CTCF) was calculated as described by Martin Fitzpatrick, University of Birmingham, United Kingdom, using the following formula: CTCF=Integrated Density-(Area of selected cell X Mean fluorescence of background readings).

### Renal Extract Preparation for Western Blot

Renal extracts from whole-kidney homogenates and NRK cells were prepared with radioimmunoprecipitation assay (RIPA) lysis buffer containing 1mM phenylmethylsulfonyl fluoride (PMSF), 1.2 mM Na_3_VO_4_, 2.5 mM NaF, and 1 mM DTT (Sigma-Aldrich, USA) and protease inhibitor cocktail (Pierce, USA). ^11^ After lysis, the extracts were centrifuged (16000 g for 20 min at 4 °C), and the supernatant was saved as the NRK cell extract. Protein concentrations were determined with the BCA Protein Assay kit (Pierce, USA). Renal extracts (20 μg) were separated by SDS-PAGE and transferred to a PVDF membrane. The membranes were incubated with antibodies to β5 subunit (1:1000; Abcam, #ab3330), α3 subunit (1:1000; Abcam, #ab119419), or β-actin (loading control, 1:1000; Sigma-Aldrich, #A5441). Probed membranes were washed three times, incubated with horseradish peroxidase-conjugated secondary antibodies (1:30,000; Seracare KPL), and assayed for enhanced chemiluminescence (Thermo Fisher Scientific, USA). Densitometry was performed with AlphaEase FC software (Alpha Innotech, USA).

### Statistical Analysis

Results are presented as the mean±standard error of the mean (SEM) (GraphPad Prism software, USA). Data (n=4–6 assays) were analyzed with a one-way ANOVA and Tukey’s posthoc test for multiple group comparisons, and an unpaired Student’s *t*-test was used when comparing differences between the means of two groups (Control *versus* CS) at a 95% level of confidence. Differences with *p*<0.05 were considered statistically significant.

## RESULTS

**Figure 1.**
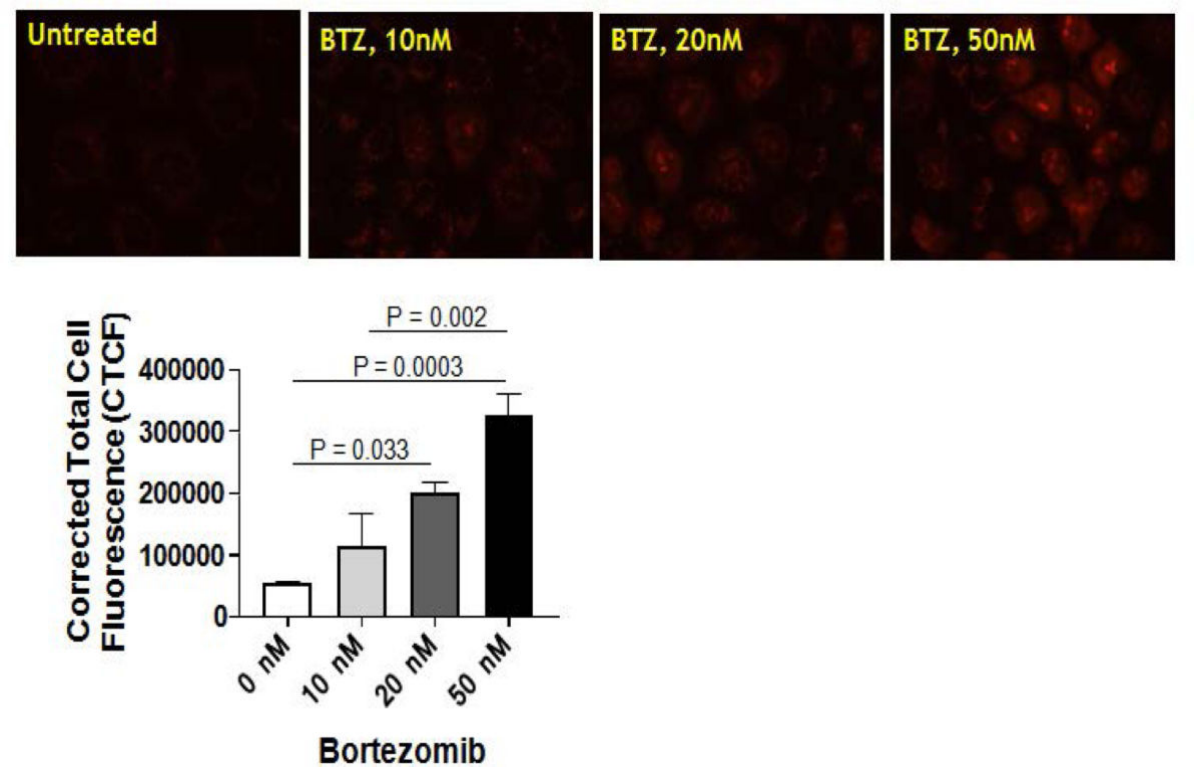
(a) Bortezomib Treatment Increases Mitochondrial Reactive Oxygen species in a Dose-dependent Manner. Normal Rat kidney Tubular (NRK) Cells were Preloaded with MitoSOX™ Redreagent Followed by Pharmacological Inhibition of the Proteasome using Bortezomib (BTZ; 0 −50 nM) for 4 hrs. DMSO Treated NRK Cells were Used as Bortezomib Treatment Increases Mitochondrial Reactive Oxygen Species in a Dose-dependent Manner. Normal rat Kidney Tubular (NRK) cells were Preloaded with Red reagent followed by Pharmacological Inhibition of the Proteasome using Bortezomib (BTZ; 0 −50 nM) for 4 hrs. DMSO Treated NRK Cells were used as Vehicle Control. Red Fluorescence was then Lisualized Using a Nikon Eclipse E800 Microscope with a Hodamine Filter Using a Water Immersion Objective (60X). All Images were Captured with Equal Exposure Times and the Fluorescence Intensity was Evaluated Using Image J Software by Calculating Corrected Total Cell Fluorescence (CTCF) (please refer to Methods section). Results are Representative of 6 Independent Analyses for Each Group and Thevalues are Expressed as the Mean±SEM (bars)(n=6). Differences Between Means were Compared with a One-way ANOVA for Multiple Group Comparisons (Vehicle, l0nM BTZ, 20n M BTZ, and 50 nM BTZ).

Bortezomib treatment increases mitochondrial ROS in NRK cells. We recently reported that Bortezomib (BTZ) treatment increases mitochondrial dysfunction and alteration of key respiratory subunits in NRK cells.^11^ This finding prompted us to determine whether BTZ treatment also increases the mitochondrial ROS. In this study, MitoSOX™ Red (Invitrogen Molecular Probes, USA) was used to detect mitochondrial ROS production in NRK cells. This modified cationic dihydroethidium dye is localized to the mitochondria where it is oxidized by superoxide to generate a bright red fluorescence.^14^ Interestingly, the mitochondrial ROS was increased after BTZ treatment of NRK cells in a dose-dependent manner (Figure 1). The vehicle control treatment had no effect on mitochondrial superoxide generation in NRK cells (Figure 1).

**Figure 2.**
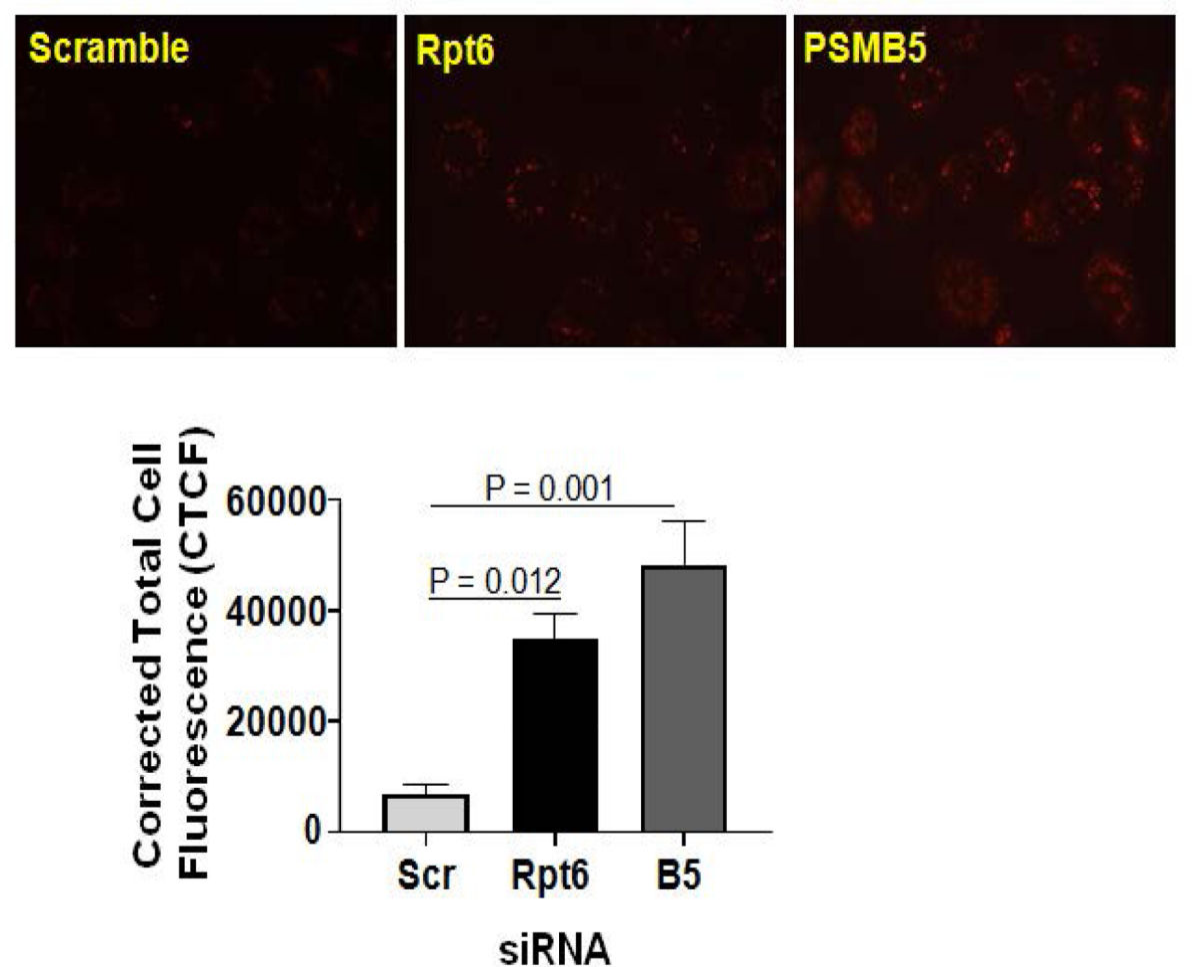
Knockdown of β5 or Rpt6 Subunit of the Proteasome Increases ROS Production in NRK Cells. Normal Rat Kidney Tubular (NRK) Cells were Preloaded with MitoSOX™ Red reagentfollowed by transfection with β5 or Rpt6 sigenome siRNA SMART Pool (100 nM). Equal Concentration of Scrambled siRNA was Used as a Control. The Next Day, Cells were Evaluated for Superoxide Production by Visualizingred Fluorescence Using a Nikon Eclipse E800 Microscope with a Rhodamine Filter Using a Water Immersion Objective (60X). All Images were Captured with Equal Exposure Times and the Fluorescence Intensity was Evaluated Using Image J Software by Calculating Corrected Total Cell Fluorescence (CTCF) (Please Refer to Methods Section). Representative Images of 4 Independent Analyses is Shown and the Values are Expressed as the Mean±SEM (bars) of (n=4). Differences Between Means were Compared with a One-way ANOVA for Multiple Group Comparisons (Scrambled siRNA, β5 siRNA, and Rpt6 siRNA Groups).

Knockdown of proteasome subunit increases mitochondrial ROS in NRK cells. Given pharmacological inhibition of the proteasome increases mitochondrial ROS production, further studies used RNA interference to assess mitochondrial ROS production in NRK cells. We found increased mitochondrial ROS production in NRK cells transfected with β5 (a 20S proteasome subunit) or Rpt6 (a 19S proteasome subunit) siRNA (Figure 2). As anticipated, scramble siRNA did not affect the mitochondrial ROS generation (Figure 2).

**Figure 3.**
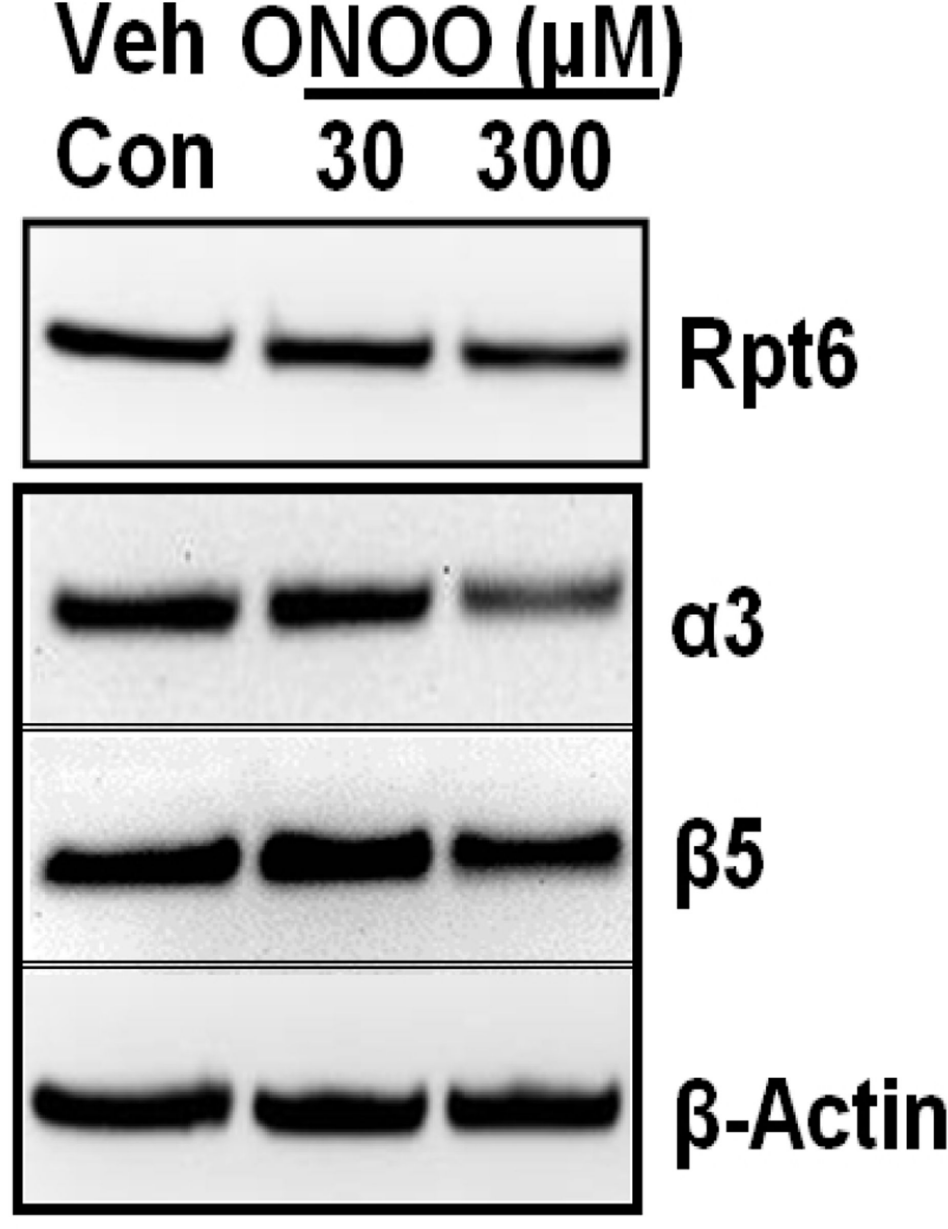
Peroxynitrite Treatment Impairs Proteasome Subunits in NRK Cells. NRK Cells were Exposed to Peroxynitrite with Dose as Indicated for 18 hrs. Cells were then Harvested and 30 μg of Renal Extracts were Evaluated with Western Blots for Proteasome Subunits (Rpt6, β5 and a3) Levels. β-actin was Used as Loading Control. Representative Western Blots of 3 Independent Analyses is Shown.

Peroxynitrite treatment altered proteasome subunit levels in NRK cells. Because we found that BTZ treatment or siRNA mediated knockdown of β5 or Rpt6 subunit increases ROS, here we attempted to evaluate whether exogenous ROS exposure alters proteasome subunit levels. We solubilized NRK cells with RIPA buffer to extract proteins and evaluated for proteasome subunits levels. Western blots of NRK cell extracts indicated decreased levels of 20S proteasome subunits (α3 and β5), after peroxynitrite treatment (Figure 3), suggesting that ROS altered these proteins levels.

## DISCUSSION

Our previous report provided evidence that the functional proteasomes are required to maintain the integrity of mitochondria in kidneys/renal cells, and the proteasome function inhibition by BTZ (in NRK cells) directly impacts the homeostasis of proteins involved with mitochondrial respiration.^11^ Here, we demonstrated that both, pharmacologic (BTZ mediated inhibition) or genetic (siRNA mediated knockdown of β5 subunit) modulation of the proteasome increases mitochondrial ROS in renal proximal tubular cells (Figures 1 and 2). Various doses of BTZ proportionately increased mitochondrial ROS (Figure 1). These studies suggest ROS as a mechanism of disrupted proteasome and mitochondrial function. *In vitro* studies using non-renal cells show induction of antioxidant enzymes following proteasome inhibition .^15–17^ In a study by Maharjan S et al, mitochondrial antioxidant (MnSOD) overexpression in Chinese hamster ovary (CHO) cells is shown to be protective against MG132 (a proteasome inhibitor)-mediated oxidative stress and cell death.^18^ Collectively, these findings suggest that proteasome appears to be involved in a redox regulation *via* antioxidant mechanisms.

Evidence from *in vitro* models (non-renal) suggests that oxidized proteins are removed by the 20S proteasome.^19–21^ ROS are considered critical determinants for proteasome function.^9,10,22^ No studies, to our knowledge, have considered the contribution of ROS on 20S proteasome subunits in renal cells. In this report, we provide evidence of the peroxynitrite-mediated decline of α3 and β5 subunits of the proteasome in NRK cells (Figure 3). These results suggest that the declined proteasome function following higher levels of ROS exposure may have resulted from the direct impairment of these subunits (β5 and α3). *In vitro* studies in mammalian cells have shown that dissociation of 20S proteasome from 19S particle occurs following ROS exposure and the levels of 26S proteasome declines with respect to higher doses of ROS.^9,10,22^ Future studies are needed to determine the mechanisms of reduction of α3 and β5subunits of the proteasome following ROS exposure.

Emerging evidence suggests that post-translational modifications to proteasome subunits may significantly impact proteasome function.^23–28^ Although we did not evaluate any post-translational protein modifications, it is plausible that α3 and β5 subunits could be the targets of oxidative post-translational modifications that alter these subunits and decrease proteasome composition and function in renal cells. It is suggested to evaluate whether post-translational modifications to β5 and α3 subunits of the proteasome reduces levels of these proteins following ROS exposure and that may provide further insight with regard to precise mechanisms of proteasome dysfunction.

## CONCLUSION

The maintenance of proteome integrity is essential for renal cell viability during stress. Misfolded or damaged proteins should be monitored by the proteasome, a protein quality-control machinery, which degrades damaged proteins. Here, we demonstrated that the decline of proteasome subunit or inhibition of proteasome subunit results in mitochondrial ROS production. On the other hand, our results show that high levels of ROS decrease the levels of key subunits of the 20S proteasome. Together these data indicate that a cycle of proteasome inhibition and ROS production that could be detrimental to renal cell health during stressful conditions, especially during renal ischemia-reperfusion injury.
